# Wiring the Vertebræ as a Means of Immobilization, Etc.*Read before the meeting of the Texas State Medical Association, and published in Transactions.

**Published:** 1891-09

**Authors:** B. E. Hadra

**Affiliations:** Galveston


### WIRING OF THE VERTEBRÆ AS A MEANS OF IMMOBILIZATION IN FRACTURE AND POTT'S DISEASE.*

B. E. HADRA, M. D., GALVESTON.

PRESENT SURGICAL interference in fractures of the spine consists in the removal of loose bones and in resection of parts of the arch, in order to free the spinal canal. The latter is done when grave symptoms indicate pressure upon the cord, or morbid changes of it, or of the dura mater. Thus, mostly older

*Read before the meeting of the Texas State Medical Association, and published in Transactions.

cases will be submitted to resection. It is my purpose to propose to you a means of adaption and retention of the broken ends before such damage is done, or before vicious consolidation has taken place.

The present apparatus of immobilization by position, extension, splints, braces, dressings, etc., is evidently sufficient only in the lightest and most tractable cases, which, unfortunately, constitute only a very small percentage. Aside from the enormous inconvenience, all these helps are ineffective when the broken ends are thoroughly separated. I remember two cases of dorsal fracture where I had the patients encased in plaster of paris, from the axilla down to the thigh. I know they were not benefited, but were made most unhappy by the treatment. It is simply impossible to keep the loosened vertebræ from gliding upon each other as long as no fixation can be had all around. Obviously, though, the contents of the thoracic and abdominal cavites can not be immobilized. Still, absolute apposition is here more a necessity than in other fractures, because, aside from giving the broken bones a chance to properly consolidate, it must be the main aim to protect the cord and the spinal nerves against pressure, twisting, and also against changes due to irritation and to extension of morbid processes from the surrounding tissues. What do we do in other fractures if the usual means do not suffice to keep the parts well adapted? We do the most natural thing in the world; we fix them to each other by direct means—clamps, nails, wires, sutures, and so on.

Now, there is no good reason why vertebral fractures should not enjoy similar advantages. There is no danger in cutting down on the vertebral column, as all operators testify, as long, of course, as the cord and the nerves remain undisturbed; and even they stand a good deal more interference that is generally conceded. It is, then, a question of feasibility and efficacy. Theoretically there should be no doubt about it. Still, practical evidence alone will satisfy nowadays. The literature—at least that at my command—offers only one case where direct fixation of broken vertebræ was planned and executed, and I think this case deserves the fullest attention. It is that of Dr. W. T. Wilkins, a short history of which is given by Prof. Keen in the "Reference Hand-book of Medical Science." It reads thus:

"I find reported a case of a child born with a hunch on its back, the mother having been severely injured the day before its birth. On operation, on the day after birth, the last dorsal and the first lumbar vertebra were found separated a half-inch, and a hernia protruded through the fissure. The spinal cord was pushed to one side. The hernia was reduced, the two vertebræ held together by a figure of 8 carbolized silk ligature, passing through the intervertebral notches of the two vertebræ, above and below, and the child was practically well in a few days."

It was my lot to test the problem in a case which was operated on last December, and I regret that I at that time did not know anything of Dr. Wilkins' case. I fastened together the spinous processes of the sixth and seventh cervical vertebra by silver-wire loops, in a case of fracture which had been acquired nearly a year before. The patient is still at the John Sealy Hospital, and I must confess is not perfectly well yet, though the efficacy of the operation has been fully demonstrated. To give a short history of the case, it may be stated that a man of 30 years of age, working as waiter in a restaurant, fell to the floor, striking with great force on his buttocks. Immediately after, he felt intense pain in his neck, and was unable to move it. On examination, the sixth cervical vertebra was found pushed forward and turned around its vertical axis to the right, whilst the spinous process of the seventh vertebra appeared unusually prominent. Patient could not open his mouth more than an inch, from what cause I could never understand. Extension was made by the head, and as the parts seemingly returned to their normal position, the neck was put in a firm cravatte. Patient, from reasons of no medical interest, left the St. Mary's Hospital a few days after his admission, and returned to his former occupation, wearing constantly his apparatus, getting along well enough with an occasional hypodermic injection of morphine. But once, when he imprudently bent his neck in a rapid and forcible way, the cravatte having been left away, he fainted, and when recovered could not stand upright. His head and neck were turned to the right, and kept perfectly stiff; right hand became numb, right arm weak; girdle pains around his upper abdomen; bladder not fully under control; slight priapism. In such condition he came to the John Sealy Hospital on November 1, 1890, ten months after the first accident. His face flushed up on the slightest provocation; his mouth could not be opened over an inch; the left upper portion of the trapezius muscle was hard and protruding, forming a tumor; his right hand colder than the left; extreme hyperæsthesia on the right side; head was rolled around to the right, and the vertebra in same position as on the first observation; muscles, though, reacted alike on both sides of the body to either current. Patient was put under chloroform, not being able to stand any manipulation without an anæsthetic. Head and upper portion of neck very movable, and crepitation distinctly heard. Reduction was easy, and the stiff cravatte was applied again. In spite of frequent adjustment and modification of the retaining apparatus, patient grew steadily worse. Pains in back, arms and around abdomen became unbearable, and walking impossible. He was in such a pitiful condition that he insisted upon any operation which would give the faintest hope of relief. I finally consented to cut down on the place of injury, which I did on the 22d day of December. My plan was to remove loose bones, if present, to sever the posterior ligaments, if they should be thickened and contracted, and finally, to wire the spinous processes, in order to steady the vertebral column.

From the improvement setting in every time the bones were well adjusted, I inferred that there was no serious change within the vertebral canal, nor in the cord itself. I therefore did not consider the opening of the canal called for. Not finding loose bones, I severed the ligamentum nuchæ and the interspinous ligaments transversely in several places, so as to expose the spinous processes fully, and also in order to remove the interference of the perhaps thickened and contracted ligaments which could have acted as an impediment to the replacement and retention of the dislocated parts, exactly as in other fractures or dislocaions. I am satisfied that this part of the operation was not only unnecessary, but that it caused all the following inflammatory symptoms, as a good deal of lacerating was unavoidable. The main aim of the operation, the wiring of the sixth and seventh spinous process, was done with silver wire, carrying it four to five times around in a figure 8. The wound which extended from the occiput down to the first dorsal vertebra was then closed, a small drainage tube inserted right over the place of wiring and the stiff cravatte reapplied. Patient did not improve for several days, but then gradually got better. After some weeks I thought that the wire had become loose, because he began to exhibit some of his former symptoms. He was put under chloroform again, the wire removed, and a new one fixed on. On this occasion it was easily seen that the lower end of the fractured spine slipped away from the upper for about one and a half inches to the right. From now on improvement went on more rapidly. Patient was able three weeks ago, that is twelve weeks after the operation, to move his head in a normal way in every direction without pain. He could open his mouth fully, walk as well as anybody else; no headache; no trouble with bladder or bowels. The right arm remained somewhat weaker, but was otherwise normal in all its functions and of normal sensation. The favorable condition, though, made me, I fear, too careless. I allowed him to be without the bandage occasionally, and removed the wire, as it kept a fistulous ulcer from closing. He became worse again, and has now considerable pain in his right arm and shoulder. The spinous process of the sixth cervical vertebra is very tender on pressure, and requires further attention, as the probable cause of the new trouble. Otherwise patient is well, and can make use of his neck without any difficulty.

This is my case, which shows that the operation is feasible and effective. But I would be a poor surgeon if I had felt satisfied with my method and the course of my case. Further experiments on the cadaver, and further reflection has led me to believe that the proposed wiring is one of the most promising, and at the same time simplest surgical procedures, provided that it be so modified as I will describe it hereafter. Of course, it is intended only for the readjustment and retention of the broken bones, therefore, in some cases it may fill all the indications; in others, it will simply be an addition to other operations.

But before proceeding I show you here a vertebral column, on which two adjoining spinous processes have been wired together in the three portions of the spine. Of course, more than two may be joined if necessary. You see how firmly the vetebrae hold together, and how resistant they become. In fact, common forces, as experienced in human life, are hardly able to undo the fixation of the two lumbar vertebræ. Of course, this method is possibly only when the spinous processes are not fractured themselves. If so, one would have to resort to the wiring of the transverse processes, as shown also on the model.

The operation consists then of the following simple acts. A good long skin incision, the center of which should be over the seat of fracture; next the muscles on either side of the spinous processes should be lifted up and drawn aside with blunt instruments, but not more than to allow one to feel the contours of the bones. Then a stout curved needle, armed with wire, is carried through the interspace between the spinous process of the broken vertebra, and that of the next upper one, as deep as possible; brought out, entered again into the next inferior interspace; brought out on the other side; entered there again into the next lower interspace; carried around the spinous process of the vetebra, below the fracture, and again carried through the middle interspace, and meeting the wire where it entered, well twisted togethef to a knot. In short, a figure of 8 loop is carried around the spinous processes of the broken vertebra and that of the next lower one, which may be repeated as often as seems advisable. In the lumbar portion of the spine simple loops will suffice, as the processes are almost horizontal. Then the wound is closed with or without drainage. Under circumstances three or even more vertebræ may be fixed together.

All this can be done in a few minutes. The operation is nearly bloodless; involves no great laceration of tissues, and can be made thoroughly aseptic. The wires are well secured in their position by the ligaments, which remain undisturbed.

More difficult is the wiring of the transverse processes. Here the muscles have to be lifted and drawn aside much more extensively. In order to avoid impending nerves in the loops, I think it would be best to do it as shown on the model; that is, first to surround one process, and then carry the thread to the next one, and again tie it here by a loop, so as to have only one wire in the interspace.

I can not resist the temptation to connect my device also with the treatment of Pott's disease. Here, too, the indication in cases where the abscess or carious bones do not call for other surgical attempts, is mainly to steady the vertebral column in order to protect the cord, to prevent the diseased bones from rubbing on each other, and finally, to make the outcome, in regard to disfigurement, as favorable as possible. Judging from my concededly limited experience, and from theoretical deductions, the proposed procedure will do better than braces, corsets, plaster jackets and the like. It seems to me called for as soon as displacement of the bones is noticed; but even if a full kyphosis should be established, it will be proper to put the patient under an anæsthetic, and to wire together the spinous processes, provided that the column can be straightened to a satisfactory extent. It occurred to me that the fixation of the spinous processes acts like a lever on the bodies of the vertebræ in front, as you can see on my model. This is a great advantage in cases where intervertebral disks are destroyed, or where cavities have formed by necrotic destruction of the vertebral bodies. Evidently such gaps would be held open, whereby better drainage would be procured, the contact of the diseased surfaces prevented, and the filling up by new tissues allowed in the most advantageous configuration. Obviously also the wiring could be added to other operations; such as establishing drainage for the abscess, or to the removal of necrotic bone, or to the trephining of the arch, etc.

Summing up, I no not claim that my proposition is an operation fitting every case of spinal fracture or of spondilitis; it is simply a method of holding the broken or diseased parts together better than any other method, and with considerably less annoyance to the patient. In many cases it may do by itself, in others it will be a desirable addition to other operations. In others again, it will be as fruitless as all other methods at our disposal. The operation is simple and free of danger, and if only a small portion of the advantages set forth could be attained, it would constitute a very desirable addition to the present means to combat such formidable and intractable ailments.

